# Bone mineral density in lower thoracic vertebra for osteoporosis diagnosis in older adults during CT lung cancer screening

**DOI:** 10.1186/s12877-024-04737-4

**Published:** 2024-03-06

**Authors:** Nandong Hu, Miaomiao Wang, Meng Yang, Xin Chen, Jiangchuan Wang, Chao Xie, Bin Zhang, Zhongqiu Wang, Xiao Chen

**Affiliations:** 1 Department of Radiology, the Affiliated Hospital of Nanjing University of Chinese Medicine, 155 Hanzhong road, 210029 Nanjing, China; 2 Department of Radiology, the Second Affiliated Hospital of Soochow University, 1055 Sanxiang road, 215004 Suzhou, China; 3 Bengbu Medical College, 2600 Donghai road, 233030 Bengbu, China; 4 Department of Radiology, Shanghai Longhua Hospital, 200032 Shanghai, China; 5 Department of Orthopaedics, University of Rochester School of Medicine, 14642 Rochester, NY USA; 6 Department of Thoracic surgery, Affiliated Hospital of Nanjing University of Chinese Medicine, Nanjing, China

**Keywords:** Quantitative computed tomography, Thoracic vertebral bone mineral density, Osteoporosis, Computed tomography, Lung cancer

## Abstract

**Background:**

Quantitative computed tomography (QCT)-based lumbar bone mineral density (LBMD) has been used to diagnose osteoporosis. This study explored the value of lower thoracic BMD (TBMD) in diagnosing osteoporosis in older adults during CT lung cancer screening.

**Methods:**

This study included 751 subjects who underwent QCT scans with both LBMD and TBMD. 141 of them was selected for a validation. Osteoporosis was diagnosed based on LBMD using the ACR criteria (gold standard). TBMD thresholds were obtained using receiver operating characteristic curve. TBMD was also translated into LBMD (TTBMD) and osteoporosis was defined based on TTBMD using ACR criteria. The performance of TBMD and TTBMD in identifying osteoporosis was determined by Kappa test. The associations between TBMD- and TTBMD-based osteoporosis and fracture were tested in 227 subjects with followed up status of spine fracture.

**Results:**

The performance of TBMD in identifying osteoporosis was low (kappa = 0.66) if using the ACR criteria. Two thresholds of TBMD for identifying osteopenia (128 mg/cm^3^) and osteoporosis (91 mg/cm^3^) were obtained with areas under the curve of 0.97 and 0.99, respectively. The performance of the identification of osteoporosis/osteopenia using the two thresholds or TTBMD both had good agreement with the gold standard (kappa = 0.78, 0.86). Similar results were observed in validation population. Osteoporosis identified using the thresholds (adjusted hazard ratio (HR) = 18.72, 95% confidence interval (CI): 5.13–68.36) or TTBMD (adjusted HR = 10.28, 95% CI: 4.22–25.08) were also associated with fractures.

**Conclusion:**

Calculating the threshold of TBMD or normalizing TBMD to LBMD are both useful in identifying osteoporosis in older adults during CT lung cancer screening.

**Supplementary Information:**

The online version contains supplementary material available at 10.1186/s12877-024-04737-4.

## Introduction

Osteoporosis is a public health issue for older adults. Dual-energy x-ray absorptiometry (DXA) is currently the criterion standard for bone mineral density (BMD) measurements. Quantitative computed tomography (QCT) can also been used to measure volume BMD. American College of Radiology (ACR) had recommended diagnostic criteria for osteoporosis based on lumbar spine QCT [1]: normal, > 120 mg/cm^3^; low bone mass, 80–120 mg/cm^3^; and osteoporosis, < 80 mg/cm^3^. QCT combined with clinical routine CT for other screening purposes allows for opportunistic screening for osteoporosis without increasing dose and scan time. A large number of chest CT scans are performed every year for early lung cancer screening [2]. The target population for chest lung cancer screening is highly overlapping with the high-risk group for osteoporosis. Usually, lumbar spine 1–2 (L1-2) are also scanned during chest CT scan. Therefore, a few studies have shown that it is feasible to opportunistically detect osteoporosis during chest CT scans [3, 4]. However, if only L1 or no part of lumbar spine is scanned during the chest scan, it is unable to evaluate whether the subjects have osteoporosis because lumbar spine BMD (LBMD) could not be measured. It would be valuable to identify strategies to use TBMD for osteoporosis evaluation.

Recent studies also used those thresholds of 120 mg/cm^3^ and 80 mg/cm^3^ to define low or very low bone mass based on thoracic vertebral bone mineral density (TBMD) [5]. However, the BMD of the thoracic spine is usually higher than that of the lumbar spine [6]. Whether thresholds of 120 mg/cm^3^ and 80 mg/cm^3^ are applicable to the thoracic spine should be validated. Interestingly, some studies have tried to use other methods for osteoporosis diagnosis based on TBMD [7, 8]. Budoff et al. [7] converted the TBMDs into equivalent LBMD values using a formula and they calculated the T score. They found that the mean T score obtained from measured LBMD was similar to that obtained from translated lumbar BMD (calculated LBMD based on TBMD). However, this study only calculated the T score and did not test the performance of the ACR criteria (80 mg/cm^3^) in translated LBMD. In addition, the BMDs of moderate levels of thoracic spines (T7-T9) were used and the mean BMDs of T10-T12 that were much closer to LBMD than to other TBMD values were not used in Budoff’s study [7].

A recent study attempted to show the thresholds for osteoporosis and osteopenia at the cervicothoracic spine using linear regression analysis [8]. They showed the threshold of osteoporosis or osteopenia in a single vertebral body (C2-T12). However, this study included subjects who received contrast-enhanced CT scans which may result in an overestimation in BMD, and it did not use phantom-based BMD. In addition, this study only showed the threshold of single vertebral body. Usually, osteoporosis diagnosis is based on the average of BMD at least two vertebral bodies [9, 10]. Moreover, the study only showed the thresholds, but the performance of those thresholds in defining osteoporosis were unknown. Studies are still needed to show the role of TBMD in osteoporosis evaluation. In the present study, we showed the thresholds of TBMD in diagnosing osteoporosis or low bone mass in older adults who underwent CT scans for lung cancer screening and validated the thresholds in an independent population. Then, we also tested our data from an independent population with outcome of incident fracture..

## Methods

### Subjects in osteoporosis evaluation

**Fig. 1:**
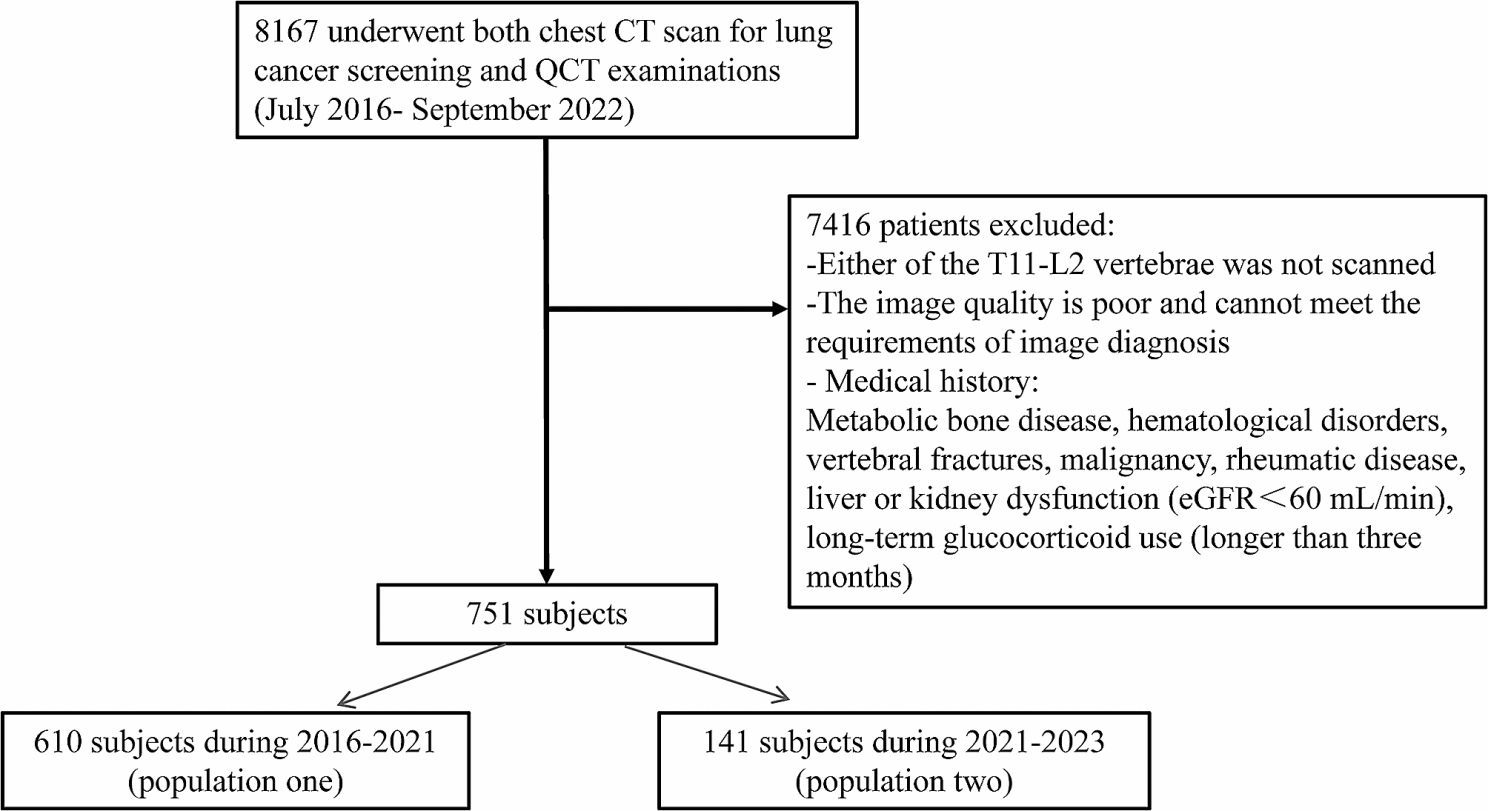
The flow chart of study population in osteoporosis analysis

This retrospective study was approved by the Institutional Ethics Review Board of our institution. Informed consent was waived because of the retrospective design. A total of 8167 subjects who underwent both chest CT scan for lung cancer screening and QCT examinations during 2016–2021 were found in our institution. Only subjects with both thoracic spine (T11-T12) and lumbar spine (L1-L2) scans were included for further analysis. Those subjects who had cancer, diabetes, chronic kidney disease, severe heart diseases, parathyroid disease, rheumatic diseases or osteoporosis on the treatment were excluded. In addition, those subjects with Genant score ≥ 1 in T11-L2 were also excluded because it would affect the BMD measurements. Finally, 610 subjects (population one) were included for osteoporosis evaluation and 141 subjects who underwent CT scans during 2021–2023 (population two) were included in a validation study. The flow chart of study population is shown in Fig. 1.

### CT scan and bone mineral density determination

All CT scans were performed using two machines (Philips Brilliance 64; GE Optima CT680). The scan protocol was as follows: tube voltage, 120 Kv; automatic mAs; and thickness of 0.625 mm, FOV of 40 mm. A total of 610 subjects (272 women and 338 men, 50–89 years old) were identified (population one). CT images were reconstructed with thickness of 1.0-1.25 mm and slice gap of 1.0 mm, and then they were transferred to the QCT workstation. Volumetric BMD determinations of the thoracic spine and lumbar spine (LBMD) were performed by using the commercially available software (Mindways QCT Pro; Mindways Software, synchronous calibration). A previous study showed that the BMD of T1-T10 was markedly higher than lumbar BMD [3]. BMD of T11 and T12 were similar and they were close to lumbar BMD [3]. Therefore, we choosing T11 and T12 as target regions to be analyzed. The illustration of BMD measurements in T11-L2 is shown in supplemental Fig. 1. The mean TBMD of T11 and T12 and LBMD of L1 and L2 were calculated for further analysis. The threshold of lumbar spine BMD recommended by the ACR was used to define osteopenia or osteoporosis as gold standard: normal, > 120 mg/cm^3^; low bone mass, 80–120 mg/cm^3^; and osteoporosis < 80 mg/cm^3^ [1]. Intra- and inter-reproducibility for BMD measurements were assessed in 60 subjects.

### Validation in patients with vertebral compression fractures

Then we showed the association between TBMD and TTBMD-based osteoporosis and vertebral compression fractures (VCF) in a longitudinal retrospective study. We searched the psychical examination system during July 2016 and January 2020. After excluding those subjects with history of malignant, rheumatic diseases, severe liver and kidney dysfunction, and vertebral fractures, a total of 7902 subjects were found to have undergone at least two or more CT scans for lung cancer screening in our institution. Seventy-two subjects with new fractures were observed. Then 212 age-, gender-, and followup time-matched subjects were selected from those subjects without new fractures during the follow-up using Propensity Score Matching at a ratio of 1:3. A total of 227 subjects (58 subjects with fracture) aged 60 years or older with QCT examinations of the thoracic spine were included for vertebral compression fractures (VCF) analysis. The flow chart of study population is shown in supplemental Fig. 2. All fractures were evaluated by CT. Volumetric TBMD determinations (T11-T12) were performed by using the commercially available software (Mindways QCT Pro; Mindways Software, asynchronous calibration) when they underwent the first CT scan. The mean TBMD of T11 and T12 was calculated. VCF was evaluated by CT based on a Genant score [11] on sagittal images or on a medical history of VCF. A new vertebral fracture was defined if a normal vertebra became deformed. The vertebrae were classified as normal (i.e., normal height) or as mild to moderate (decrease in height of approximately 20–40%), or severely deformed (decrease in height of more than 40%).

### Statistical analysis

The comparison between TBMD and LBMD was performed by using the paired t test.The correlation between TBMD and LBMD was analyzed using Pearson’s correlation and linear regression analyses. A receiver operating characteristic (ROC) curve was used to determine the threshold of TBMD (Youden index) in defining osteopenia or osteoporosis and was also used to show the performance of osteoporosis in predicting fracture. The agreement of different methods in identifying osteopenia or osteoporosis was compared by using the Kappa method. A Bland-Altman plot was used to show the intra- and inter-reproducibility among readers. Linear regression analyses were also used to define the threshold of TBMD by substituting the variable of 120 mg/cm^3^ and 80 mg/cm^3^ into linear regression equation. A Cox proportional hazards model was applied to show the association between baseline osteoporosis and VCF in population two. Statistical significance was defined as *p* < 0.05.

## Results

### Characteristics of subjects

**Fig. 2:**
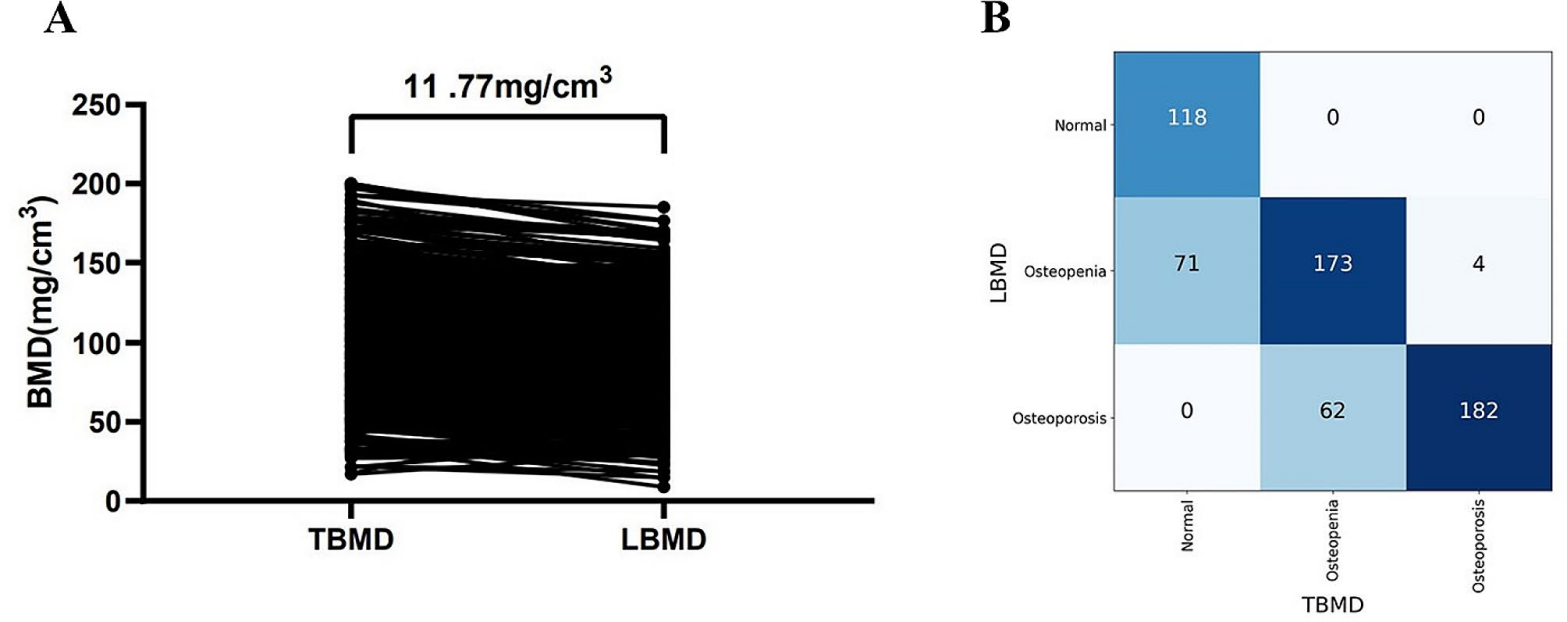
The difference between thoracic vertebral bone mineral density (TBMD) and lumbar bone mineral density (LBMD) (**A**) and the performance of thoracic vertebral bone mineral density (TBMD) in identifying osteopenia and osteoporosis based one ACR criteria (**B**) in population one. Kappa test showed moderate agreement in identifying osteoporosis between TBMD and lumbar spine BMD (kappa = 0.66) (**B**)

The characteristics of subjects are shown in Table 1. The mean age was 68.2 years old (women, 67.0; men, 69.2) for population one. The average LBMD was lower than the TBMD (mean differences = 11.8 mg/cm^3^, 95% confidence interval = -12.5 to -11.0, *p* < 0.01) (Fig. 2A). The BMDs of T11/T12 and lumbar spine (L1-L2) were 102.2 ± 36.1 mg/cm^3^ and 90.43 ± 33.5 mg/cm^3^, respectively. We also separately showed the TBMD based on gender in our populations (supplemental Fig. 3). The TBMDs of women were slightly lower than that in men, but no significant differences were observed (*p* > 0.05). The Bland-Altman analysis showed a good reproducibility of TBMD and LBMD measurements among readers (supplemental Fig. 4).

**Table 1:** Characteristics of subjects

	Population one (*n* = 610)	Population two (*n* = 141)	Population three (*n* = 227)
Age (years)	68.2 ± 11.01	64.1 ± 8.47	73.6 ± 8.78
Gender (men/women)	338/272	73/68	52/175
BMI (kg/m2)	24.24 ± 2.77	23.76 ± 2.63	22.32 ± 2.56
LBMD (mg/cm3)	90.43 ± 33.47	116.50 ± 35.96	/
TBMD (mg/cm3)	102.20 ± 35.84	125.40 ± 32.95	109.6 ± 28.40
Osteoporosis (n)	244	20	

Population one was included for osteoporosis assay using low thoracic vertebral bone mineral density (TBMD). Population two were included for a validation. Population three was included to test the role of TBMD in predicting fracture

BMI: Body mass index; LBMD: lumbar bone mineral density

The mean age was 64.5 years old (women, 62.8; men, 64.9) for population two. The average LBMD was lower than the TBMD (mean differences = 8.9 mg/cm^3^, 95% confidence interval = -10.8 to -7.0, *p* < 0.01). The mean BMDs of T11/T12 and lumbar spine (L1-L2) were 125.2 ± 32.8 mg/cm^3^ and 116.5 ± 36.0 mg/cm^3^, respectively.

### Diagnostic performance of TBMD for osteoporosis based on ACR criteria

First, we used 120 mg/cm^3^ and 80 mg/cm^3^ which were the ACR criteria for osteoporosis diagnosis based on LBMD for TBMD to define osteopenia and osteoporosis in population one. The agreement of osteopenia and osteoporosis identification between TBMD and LBMD was moderate (kappa = 0.66, 95% confidence interval (CI): 0.61–0.71), *p* < 0.001) based on the ACR diagnostic criteria (Fig. 2B). The positive predictive value (PPV) and specificity were high, but the negative predictive value (NPV) and sensitivity were low (Table 2). Only 74.0% (182/244) of osteoporosis subjects were diagnosed with osteoporosis when the 80 mg/cm^3^ threshold was used for TBMD. Similar results were observed in population two (Table 2).

**Table 2:** Performance of low thoracic vertebral bone mineral density (TBMD)

		Osteopenia + Osteoporosis					Osteoporosis					Osteopenia				
		Accuracy	PPV	NPV	Se	Sp	Accuracy	PPV	NPV	Se	Sp	Accuracy	PPV	NPV	Se	Sp
Population one	TBMD^a^	88.4%	100.0%	62.4%	85.6%	100.0%	89.3%	97.9%	85.6%	75.0%	98.9%	77.5%	73.6%	80.0%	69.8%	82.9%
	TBMD^b^	93.3%	98.9%	75.8%	92.7%	95.8%	94.3%	92.3%	95.3%	93.0%	95.1	87.4%	89.4%	86.2%	78.2%	93.6%
	TTBMD	94.3%	96.2%	86.1%	96.7%	83.9%	94.1%	95.0%	95.8%	93.9%	94.3%	88.4%	86.1%	89.9%	85.1%	90.6%
Population Two	TBMD^a^	77.3%	96.7%	63.0%	65.9%	96.2%	93.6%	100.0%	93.1%	55.0%	100%	76.6%	95.0%	70.3%	55.9%	97.3%
	TBMD^b^	90.8%	92.3%	88.5%	92.1%	86.8%	99.2%	100%	99.2%	95.0%	99.2	90.1%	89.9%	91.7%	91.2%	90.4%
	TTBMD	86.5%	80.7%	96.2%	97.3%	75.0%	98.6%	95.0%	99.2%	95.0%	99.2%	86.5%	96.2%	80.9%	74.6%	97.3%

PPV: positive prediction value; NPV: negative positive value; Se: sensitivity; Sp: specificity

a based on the thresholds of 120 mg/cm^3^ and 80 mg/cm^3^

b based on the thresholds of 128 mg/cm^3^ and 91 mg/cm^3^

### The threshold of TBMD for osteoporosis evaluation

**Fig. 3:**
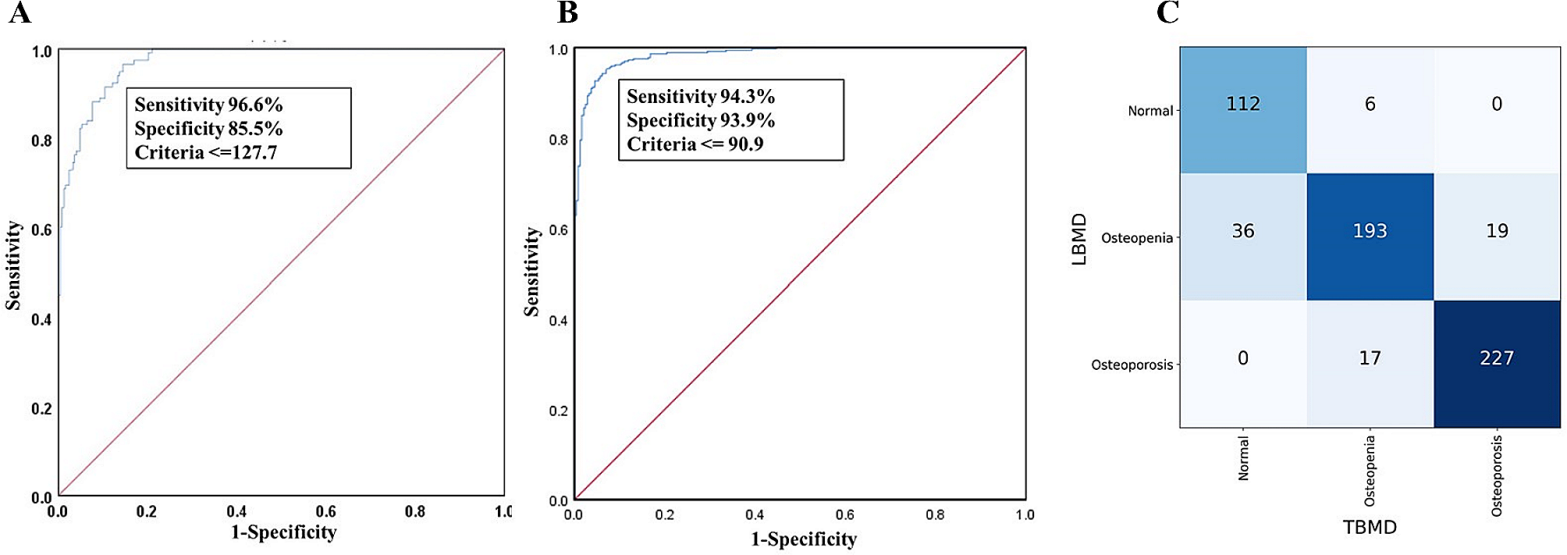
The performance of thoracic vertebral bone mineral density (TBMD) in identifying osteopenia and osteoporosis in population one. Receiver operating characteristic (ROC) curve was calculated for identification of osteopenia (**A**) and osteoporosis (**B**). The threshold was 128 mg/cm^3^ and 91 mg/cm^3^. Then we identified osteopenia and osteoporosis using the two thresholds. Kappa test showed good agreement in identifying osteoporosis between TBMD and lumbar spine BMD (kappa = 0.80) (**C**)

Subsequently, we calculated the thresholds of TBMD using ROC curves in population one. Continuous TBMD data had acceptable performance in identifying osteopenia (127.7 mg/cm^3^, area under the curve (AUC) = 0.97, with a sensitivity of 96.6%, specificity of 85.5%) (Fig. 3A) or osteoporosis (90.9 mg/cm^3^, AUC = 0.99, with a sensitivity of 94.3%, specificity of 93.9%) (Fig. 3B). We obtained the threshold of TBMD in identifying osteopenia (128 mg/cm^3^) and osteoporosis (91 mg/cm^3^) using the LBMD ACR definition as the reference. Then, we defined osteopenia and osteoporosis based on the two thresholds of TBMD. The agreement between TBMD and LBMD was high (kappa = 0.80, 95% CI: 0.76–0.84, *p* < 0.001) (Fig. 3C) in osteopenia and osteoporosis identification. Subgroup analysis based on age (≥ 65 or < 65 years), gender and body mass index (≥ 24 or < 24 kg/m^2^) showed similar trends (supplemental Tables 1–3). The accuracy of TBMD was 94.9% (112/118), 77.8% (193/248) and 93.0% (227/244) in identifying normal, osteopenia, and osteoporosis, respectively. The negative positive value (NPV) and sensitivity were higher than those using the thresholds of TBMD at 120 mg/cm^3^ and 80 mg/cm^3^, respectively (Table 2).

Then we test the performance of 128 mg/cm^3^ and 91 mg/cm^3^ in identifying osteopenia and osteoporosis in population two. The agreement between TBMD and LBMD was high (kappa = 0.84, 95% CI: 0.76–0.91, *p* < 0.001) (supplemental Fig. 5) in osteopenia and osteoporosis identification. 46 of 53 normal BMD and 19 of 20 osteoporosis was correctly identified. We also calculated the sensitivity, specificity, positive prediction value (PPV), NPV of the obtained thresholds for osteoporosis evaluation in population two (Table 2). Similarly, the NPV and sensitivity were higher than those using the thresholds of TBMD at 120 mg/cm^3^ and 80 mg/cm^3^ identifying osteopenia and osteoporosis.

### Normalized TBMD for osteoporosis evaluation

**Fig. 4:**
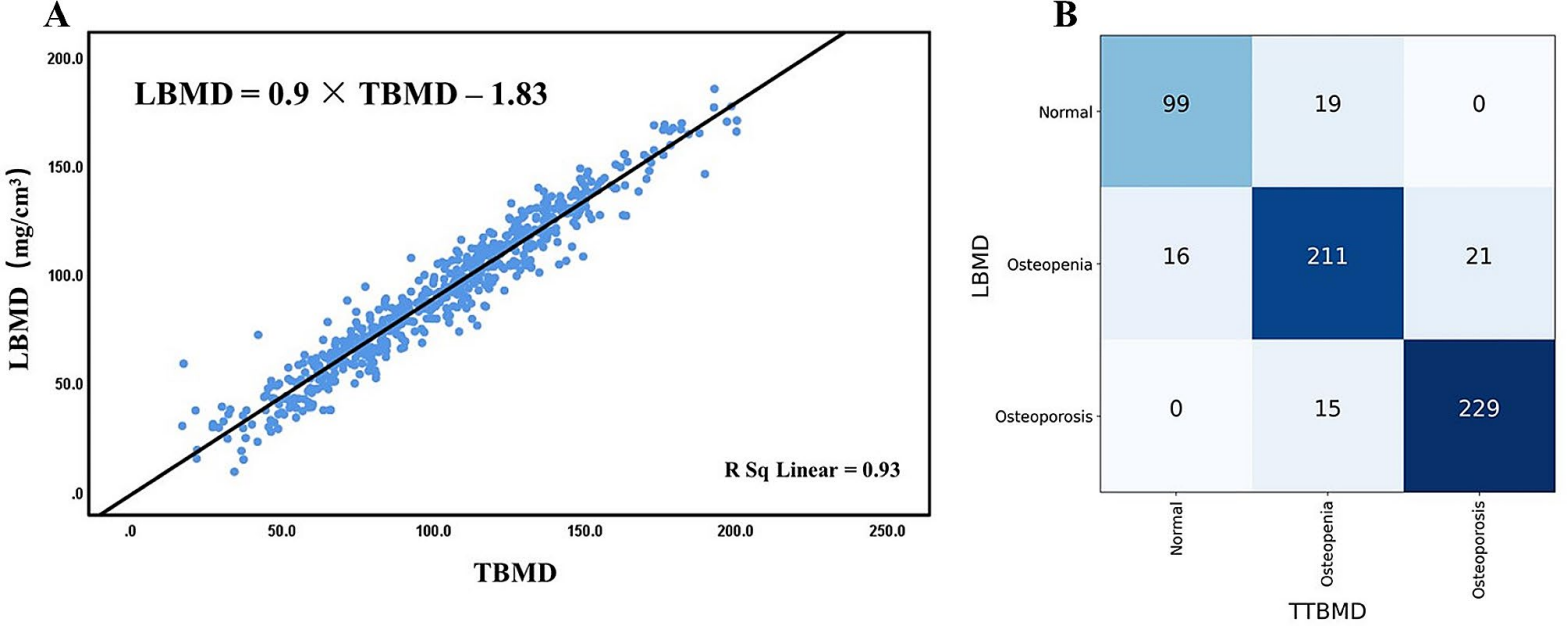
A formula (LBMD = 0.9 × TBMD– 1.83) was obtained to translate lumbar bone mineral density (LBMD) based on thoracic BMD (TBMD) (**A**) and the performance of translated LBMD in identifying osteopenia and osteoporosis. Kappa test showed good agreement in identifying osteoporosis between translated LBMD (TTBMD) and LBMD (kappa = 0.82) (**B**)

We also found that LBMD was positively correlated with TBMD (*r* = 0.93) (Fig. 4A). We next normalized TBMD to LBMD using the following formula: LBMD = 0.9 × TBMD − 1.83 based on data of population one. Then, we obtained the translated LBMD (TTBMD) based on the TBMD data. Using the ACR criteria, we defined osteopenia and osteoporosis based on the TTBMD. The agreement of the osteopenia and osteoporosis identification between LBMD and the TTBMD was high (kappa = 0.82, 95% CI: 0.78–0.86, *p* < 0.001) (Fig. 4B). Subgroup analysis based on age (≥ 65 or < 65 years), gender and body mass index (≥ 24 or < 24 kg/m^2^) showed similar trends (supplemental Tables 1–3). The accuracy of TTBMD was 83.9% (99/118), 85.1% (211/248) and 94.0% (229/244) in identifying normal vertebrae, osteopenia, and osteoporosis, respectively. The NPV and sensitivity were higher than those using the thresholds of TBMD at 120 mg/cm^3^ and 80 mg/cm^3^, respectively (Table 2).

Then we further test the performance TTBMD in identifying osteopenia and osteoporosis in population two. The agreement of the osteopenia and osteoporosis identification between LBMD and the TTBMD was high (kappa = 0.80, 95% CI: 0.62–0.88, *p* < 0.001) (supplemental Fig. 6). 51 of 53 normal and 19 of 20 osteoporosis was correctly identified. In addition, the NPV and sensitivity were higher than those using the thresholds of TBMD at 120 mg/cm^3^ and 80 mg/cm^3^ in identifying osteopenia and osteoporosis (Table 2).

### The association between TBMD and TTBMD defined osteoporosis and vertebral fractures

**Fig. 5:**
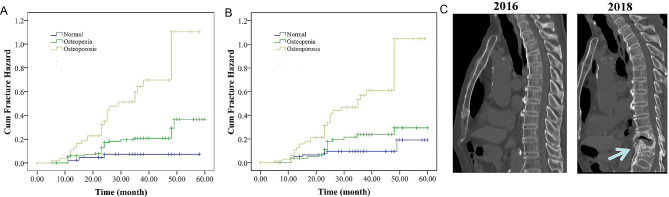
The association between low bone mass and vertebral compression fracture. Cumulative hazards of vertebral compression fracture stratified by osteopenia and osteoporosis based on threshold method (**A**) and translated thoracic vertebral bone mineral density (**B**), and one case of fracture in subjects with thoracic vertebral bone mineral density (TBMD) of 89 mg/cm^3^ at baseline (**C**). A: normal: TBMD > 128 mg/cm^3^; osteopenia: TBMD 91–128 mg/cm^3^; osteoporosis: TBMD < 91 mg/cm^3^. B: normal: TTBMD > 120 mg/cm^3^; osteopenia: TBMD 80–120 mg/cm^3^; osteoporosis: TBMD < 80 mg/cm^3^. C: A 77 year-old man with a TBMD of 89 mg/cm^3^ at 2016. Two years later, vertebral compression fractures occurred at T11 (Arrow)

In addition, we also showed the association between TBMD and TTBMD defined osteoporosis and vertebral fractures in population three. The median follow-up was two years (range 1–4 years). The characteristics of subjects are shown in Table 1. The mean age was 73.6 years old. Sixty cases of osteoporosis were observed using the threshold of 91 mg/cm^3^. Fifty-eight cases of fracture (45 women and 13 men) were recorded during the follow-up. Using 91 mg/cm^3^ as a threshold, 35 osteoporosis cases were observed in 58 cases of fractures (60.3%). Cox regression analysis also showed that the risk of fractures was associated with osteopenia (adjusted hazard ratio (HR) = 4.43, 95% confidence interval (CI): 1.31–15.06) and osteoporosis (adjusted hazard ratio (HR) = 18.72, 95% CI: 5.13–68.36). The cumulative risk curve is shown in Fig. 5A. Similar association was observed between TBMD cutoff point-based osteoporosis and severe VCF (supplemental Fig. 7A).

Subsequently, we also calculated the TTBMD using above equation (LBMD = 0.9 × TBMD − 1.83). Then we used 80 mg/cm^3^ as the threshold to define osteoporosis. Sixty-nine cases of osteoporosis were observed. Thirty-four osteoporosis cases were observed in 58 cases of fractures (58.6%). Cox regression analysis also showed that osteopenia (adjusted HR = 2.78, 95% CI: 1.16–6.72) and osteoporosis (adjusted HR = 10.28, 95% CI: 4.22–25.08) were associated with the risk of fractures. The cumulative risk curve is shown in Fig. 5B. Similar association was observed between TTBMD-based osteoporosis and severe VCF (supplemental Fig. 7B). One case of fracture is showed in subjects with baseline TBMD of 89 mg/cm^3^ (Fig. 5C).

## Discussion

The thoracic spine is usually scanned during chest CT examinations, which could supply the opportunity for spine BMD measurement. However, the ACR recommended criteria are based on LBMD. The thresholds of TBMD for osteoporosis diagnosis have not been clarified. In the present study, we explored two approaches to define low bone mass or osteoporosis based on TBMD. Our data showed that the diagnosis of osteopenia or osteoporosis using TBMD had good agreement with LBMD. Two methods could be used. One is to calculate the threshold of TBMD in identifying osteopenia or osteoporosis. The other is to normalize the data of TBMD to LBMD, and then use the ACR criteria to define osteopenia or osteoporosis. The two methods showed good agreement with that based on LBMD.

TBMD is usually higher than LBMD [4, 7, 12]. In addition, TBMD is highly correlated with LBMD (*r* > 0.80) [7, 13] Our data also showed higher correlation coefficients (*r* = 0.93). The highly positive correlation makes it feasible to normalize TBMD to LBMD for osteoporosis definition. Our data showed that the performance of osteoporosis identification was good using the normalized LBMD. A previous study also showed a comparable T-score results between measured BMD and translated LBMD [7].

Few studies have shown the thresholds of lower thoracic vertebral BMD in defining osteopenia or osteoporosis [8]. Therefore, we also calculated the threshold of TBMD in identifying osteopenia and osteoporosis using the LBMD ACR definition as the reference. The cutoff values were 127.7 mg/cm^3^ for osteopenia and 90.9 mg/cm^3^ for osteoporosis. The two thresholds showed good agreement with LBMD in defining osteopenia or osteoporosis (kappa = 0.80). Interestingly, Rühling et al. reported BMD thresholds at T11 (127.8 mg/cm^3^ for osteopenia and 91.4 mg/cm^3^ for osteoporosis) and T12 (121.0 mg/cm^3^ for osteopenia and 83.5 mg/cm^3^ for osteoporosis) for the identification of osteopenia and osteoporosis using linear regression analysis [8]. Unfortunately, the diagnostic performance was not shown in their study, and they did not show the thresholds of combined T11 and T12. In Rühling et al.’s study [8], a linear regression equation was obtained between T11/T12 and L1-L3. Then, the thresholds were calculated based on these equations and the ACR criteria (120 mg/cm^3^ and 80 mg/cm^3^). We calculated the thresholds using the ROC curve which has been widely used to choose the optimal cutoff value. On the whole, a previous study [8] and our data indicated that ACR criteria may be not fit for TBMD in identifying osteoporosis. Although our study obtained two thresholds that were close to a recent report (127.8 mg/cm^3^ for osteopenia and 91.4 mg/cm^3^ for osteoporosis) [8], many efforts should be undertaken to define a generalizable TBMD threshold for osteoporosis. Because of the relative small sample size, further studies with a larger sample sizes are needed. Our study is an exploration.

Osteoporosis is associated with bone fractures [14]. We also used the thresholds of TBMD (91 mg/cm^3^) and TTBMD to predict the outcomes of incident fracture in an independent population in this study. Osteopenia and osteoporosis defined by the thresholds or the TTBMD were associated with the risk of VCF, indicating that our methods are credible. Our data showed that approximately 60.3–58.6% of subjects with VCF had osteoporosis. VCF is also associated with muscle quality [15, 16] or early menopause and current smoking [17]. Previous data showed that osteoporosis accounts for approximately half of all hip fractures [18]. Our data showed a slightly better performance than the previous study [18]. Those data considering clinical endpoints, such as fracture, further indicated that the ACR criterion of 80 mg/cm^3^ was not fit for TBMD. A new threshold should be set for TBMD in defining osteoporosis.

Lung cancer screening with low-dose CT has been gradually increased over the past decade. The U.S. Preventive Services Task Force (USPSTF) recommends that adults aged 50 to 80 years old who have a 30 pack-year smoking history and currently smoke or have quit within the past 15 years should undergo annual screening for lung cancer [19]. Tobacco use in China is a critical public health issue. There are more than 300 million smokers and 740 million nonsmokers exposed to second-hand smoke in China [20]. Moreover, lung caner screening is recommended for subjects with chronic obstructive pulmoriary disease China [21]. Screening is also high in women because the incidence of lung cancer in women and nonsmokers is high in China [22]. Lung cancer is the leading cause of cancer-related deaths in China [23]. Therefore, a large population undergoes lung cancer screening every year in China. Old age is a common risk factor for osteoporosis and lung cancer. Osteoporosis evaluation during CT scans for lung cancer screening does not cause additional radiation exposure, cost or increase scanning time. Some studies have shown that cardiac CT is useful to identify individuals with low BMD and individuals with a high risk of fracture [3, 24]. Our study first reported that TBMD based on CT for lung cancer screening is also useful to identify individuals with low BMD and individuals with a high risk of fracture.

The ACR criteria for osteoporosis diagnosis do not consider the sex differences [2]. In this study, we also did not separately calculate the TBMD threshold in women and men. A recent study showed that there were no significant differences in TBMD between men and women in old age (62.2 ± 12.1 years), which was very close to our population study [24]. Budoff et al. also reported similar results between men and women aged 50–75 years old [7]. Our data showed that the TBMD of women was lower than that of men. However, no significant differences were found in our populations. These results indicated that the thresholds of TBMD obtained in our study were fit for both men and women.

Our study had several advantages. First, we validated the obtained thresholds in another independent population and in a fracture cohort. Second, we used two methods to define osteoporosis based on lower thoracic vertebral BMD. Third, we also validated our results in a population with VCF. Our study also had limitations. First, the sample size was not large in calculating the threshold of TBMD or translating TBMD into LBMD. Our results might need to be validated in studies with large sample size or in an independent population. Second, only lower thoracic spine (T11-T12) were included in osteoporosis analysis because the two thoracic spine locations were close to the lumbar spine, and the mean BMD of the two thoracic spine location was the lowest in thoracic spine [24]. The thresholds in our study only apply to the mean BMD of T11-T12 because volumetric BMD in vertebrae increases at increasingly higher levels. Third, all our subjects were older than 50 years old. The sample size was small for young adults because BMD measurement was usually performed on old adults. Whether our results can be generalized to younger adults will also require further exploration. A recent study showed that the BMD of T11 and T12 were higher than 150 mg/cm^3^ in subjects < 50 years old [8]. The addition of young adults may not change our results. Finally, it would be better to analyze non-vertebral fracture too. However, the information on history of non-vertebral fracture was missing in some subjects.

In conclusion, our study showed that the ACR criteria might not be suitable for TBMD in defining osteoporosis. However, BMD based on the lower thoracic spine can be used to identify osteopenia and osteoporosis if a slightly higher threshold or TTBMD is used. Two approaches, based on TBMD threshold and data regarding translated LBMD, both showed good diagnostic performance in identifying osteoporosis. TBMD can also predict the incident new vertebral fractures. More studies are needed to obtain generalizable TBMD thresholds for osteoporosis.

### Electronic supplementary material

Below is the link to the electronic supplementary material.

Supplementary Material 1

## Data Availability

All data and materials generated or analyzed during this study are not publicly available but are available from the corresponding author on reasonable request.

## Abbreviations

ACR: American College of Radiology
AUC: Area under the curve
BMD: Bone mineral density
CT: Computed tomography
HR: Hazard ratio
HU: Hounsfield units
ROC: Receiver operating characteristic
VCF: Vertebral compression fractures
