# Supplementary material for: Bone mineral density in lower thoracic vertebra for osteoporosis diagnosis in older adults during CT lung cancer screening
Source: BMC Geriatr. 2024 Mar 6;24:237. doi: 10.1186/s12877-024-04737-4 (PMC10918915; doi:10.1186/s12877-024-04737-4)

**Bone mineral density in lower thoracic vertebra for osteoporosis diagnosis in older adults during CT lung cancer screening**

**Running head: Thoracic vertebral bone mineral density for osteoporosis diagnosis**

Nandong Hu^1^, Miaomiao Wang^1^, Meng Yang^2^, Xin Chen^3^, Jiangchuan Wang^1^, Chao Xie^4^, Bin Zhang^5^, Zhongqiu Wang^1^, Xiao Chen^1^

1.Department of Radiology, the Affiliated Hospital of Nanjing University of Chinese Medicine, 155 Hanzhong road, Nanjing 210029, China

2.Bengbu Medical College, 2600 Donghai raod, Bengbu 233030, China

3.Department of Radiology, Shanghai Longhua Hospital, Shanghai 200032, China

4.Department of Orthopaedics, University of Rochester School of Medicine, NY 14642, USA

5.Department of Thoracic surgery, Affiliated Hospital of Nanjing University of Chinese Medicine

Nandong Hu, Miaomiao Wang and Meng Yang contributed equally to this work.

Corresponding author: Xiao Chen (ORCID ID, 0000-0002-8354-9087), Department of Radiology, the Affiliated Hospital of Nanjing University of Chinese Medicine, 155 Hanzhong road, Nanjing 210029, China Tel: +86 025 86617141, Email: [fsyy00597@njucm.edu.cn;](mailto:fsyy00597@njucm.edu.cn;)

**Supplemental Table 1** Subgroup analysis for the performance of TBMD and TTBMD in identifying osteoporosis based on gender in population one

|  | LBMD | | | |
| --- | --- | --- | --- | --- |
|  | Male | | Female | |
|  | Kappa | P | Kappa | P |
| TBMD | 0.764 | < 0.001 | 0.845 | < 0.001 |
| TTBMD | 0.773 | < 0.001 | 0.866 | < 0.001 |

TBMD:thoracic bone mineral density; TTBMD: translated TBMD

TBMD based on the thresholds of 128 mg/cm^3^ and 91 mg/cm^3^

TTBMD based on the thresholds of 120 mg/cm^3^ and 80 mg/cm^3^

TBMD:thoracic vertebral bone mineral density; TTBMD: translated lumbar BMD (TTBMD) based on the TBMD data

**Supplemental Table 2** Subgroup analysis for the performance of TBMD and TTBMD in identifying osteoporosis based on age in population one

|  | LBMD | | | |
| --- | --- | --- | --- | --- |
| Age (years) | ≤ 65 (n = 264) | | > 65 (n = 346) | |
|  | Kappa | P | Kappa | P |
| TBMD | 0.763 | < 0.001 | 0.781 | < 0.001 |
| TTBMD | 0.790 | < 0.001 | 0.793 | < 0.001 |

TBMD:thoracic bone mineral density; TTBMD: translated TBMD

TBMD based on the thresholds of 128 mg/cm^3^ and 91 mg/cm^3^

TTBMD based on the thresholds of 120 mg/cm^3^ and 80 mg/cm^3^

**Supplemental Table 3** Subgroup analysis for the performance of TBMD and TTBMD in identifying osteoporosis based on body mass index in population one

|  | LBMD | | | |
| --- | --- | --- | --- | --- |
| Age (years) | ≤ 24 (n = 372) | | > 24 (n = 238) | |
|  | Kappa | P | Kappa | P |
| TBMD | 0.786 | < 0.001 | 0.792 | < 0.001 |
| TTBMD | 0.794 | < 0.001 | 0.805 | < 0.001 |

TBMD:thoracic bone mineral density; TTBMD: translated TBMD

TBMD based on the thresholds of 128 mg/cm^3^ and 91 mg/cm^3^

TTBMD based on the thresholds of 120 mg/cm^3^ and 80 mg/cm^3^

**Supplemental Figure 1.** The illustration of bone mineral density measurements in thoracic spine (T11-T12) and lumbar spine (L1-L2). a-d: axial images of T11, T12, L1, L2 vertebral bodies; e-h: Sagittal images of T11, T12, L1, L2 vertebral bodies; i-l: Coronal images of T11, T12, L1, L2 vertebral bodies


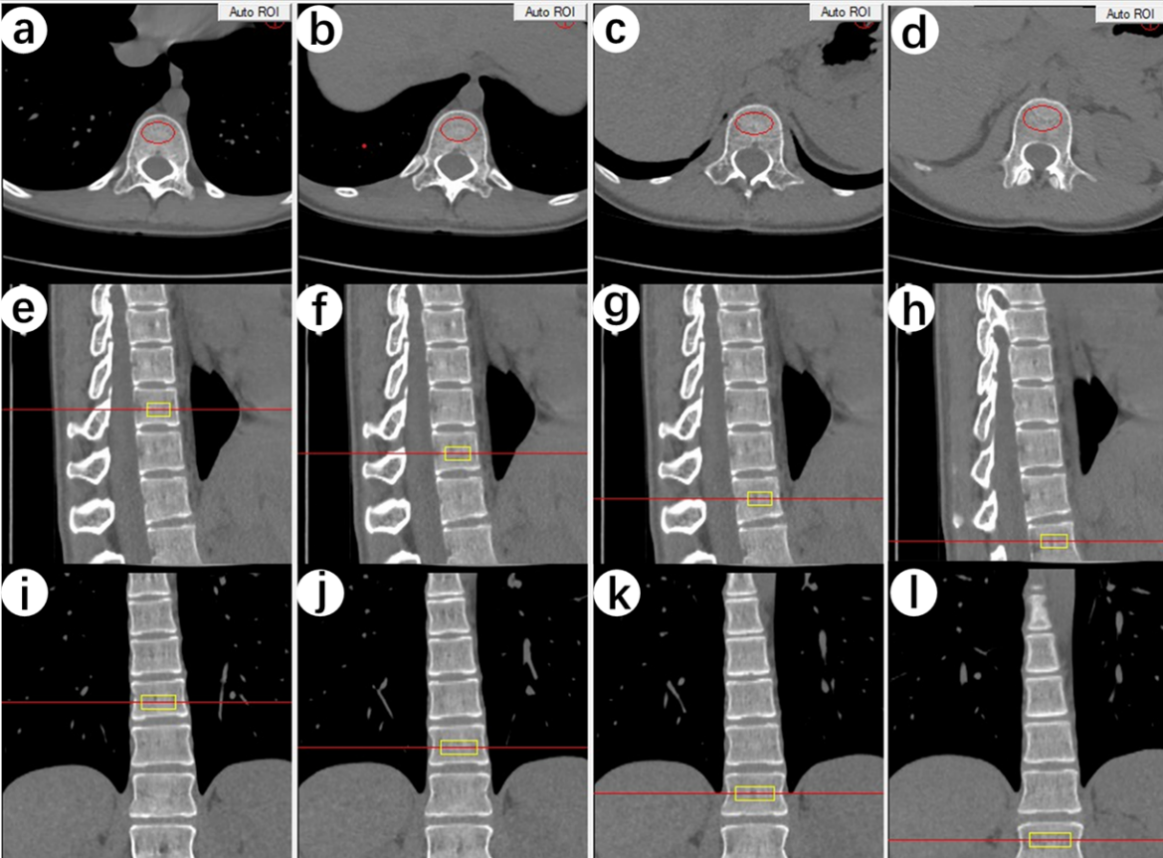


**Supplemental Figure 2.** The flow chart of study population three. PSM: Propensity Score Matching.


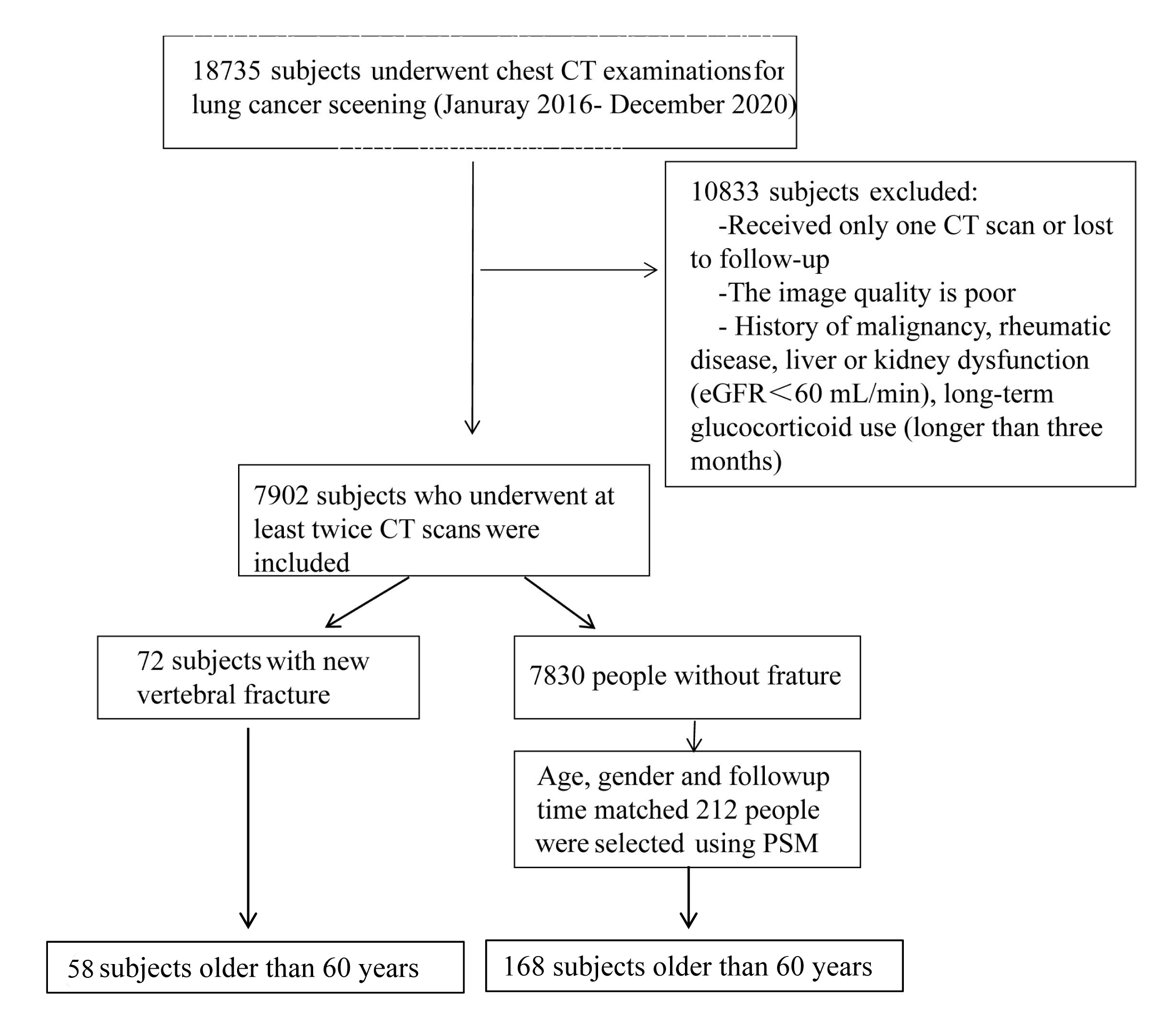


**Supplemental Figure 3.** The differences in lower thoracic vertebral bone mineral density (T11 and T12) and lumbar bone mineral density (LBMD) between men and women in population one.


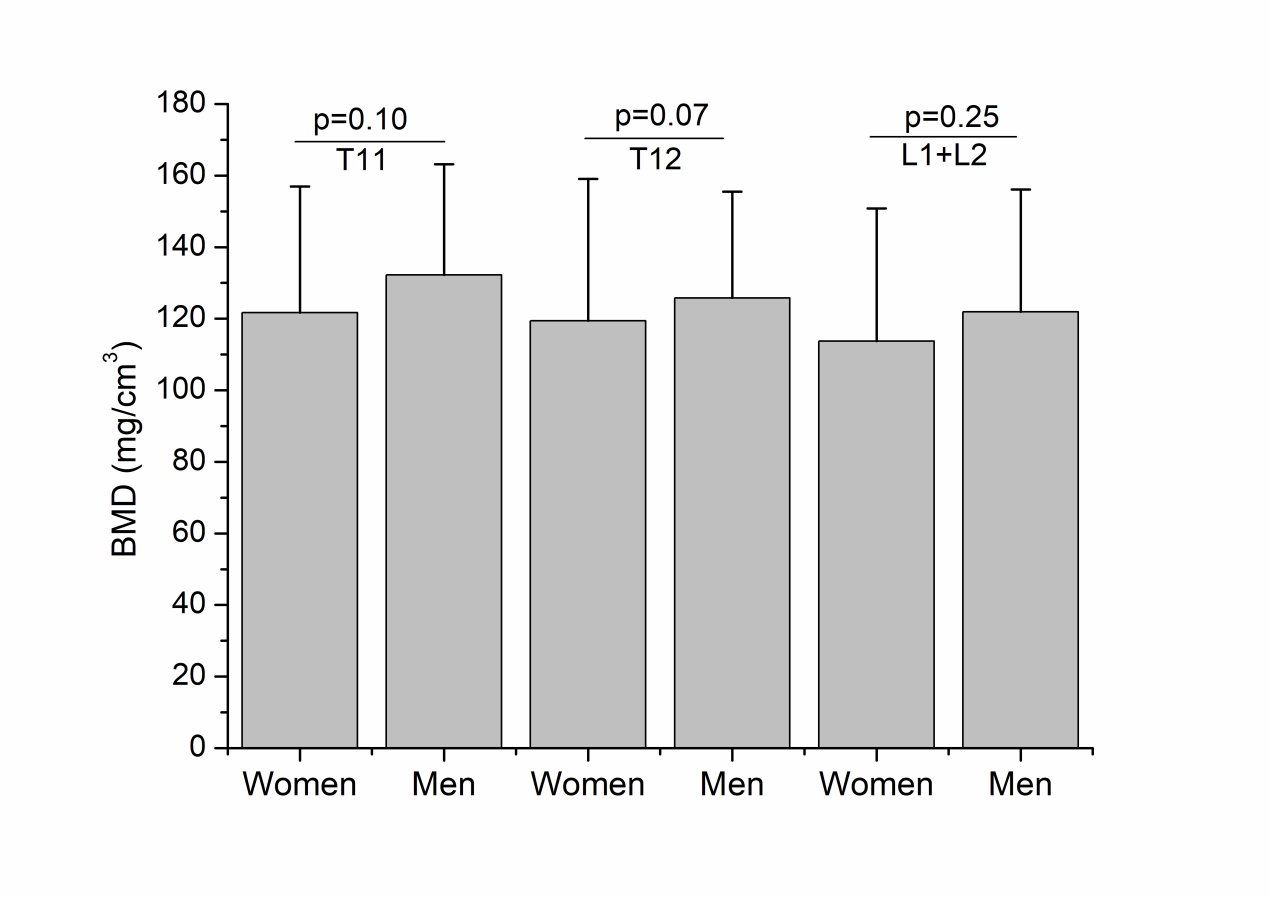


**Supplemental Figure 4.** The Bland-Altman analysis showed a good reproducibility of thoracic bone mineral density (TBMD, mean T11 and T12) and lumbar BMD (LBMD, mean L1 and L2) measurements among readers. A: Intra--reproducibility; B: inter-reproducibility.


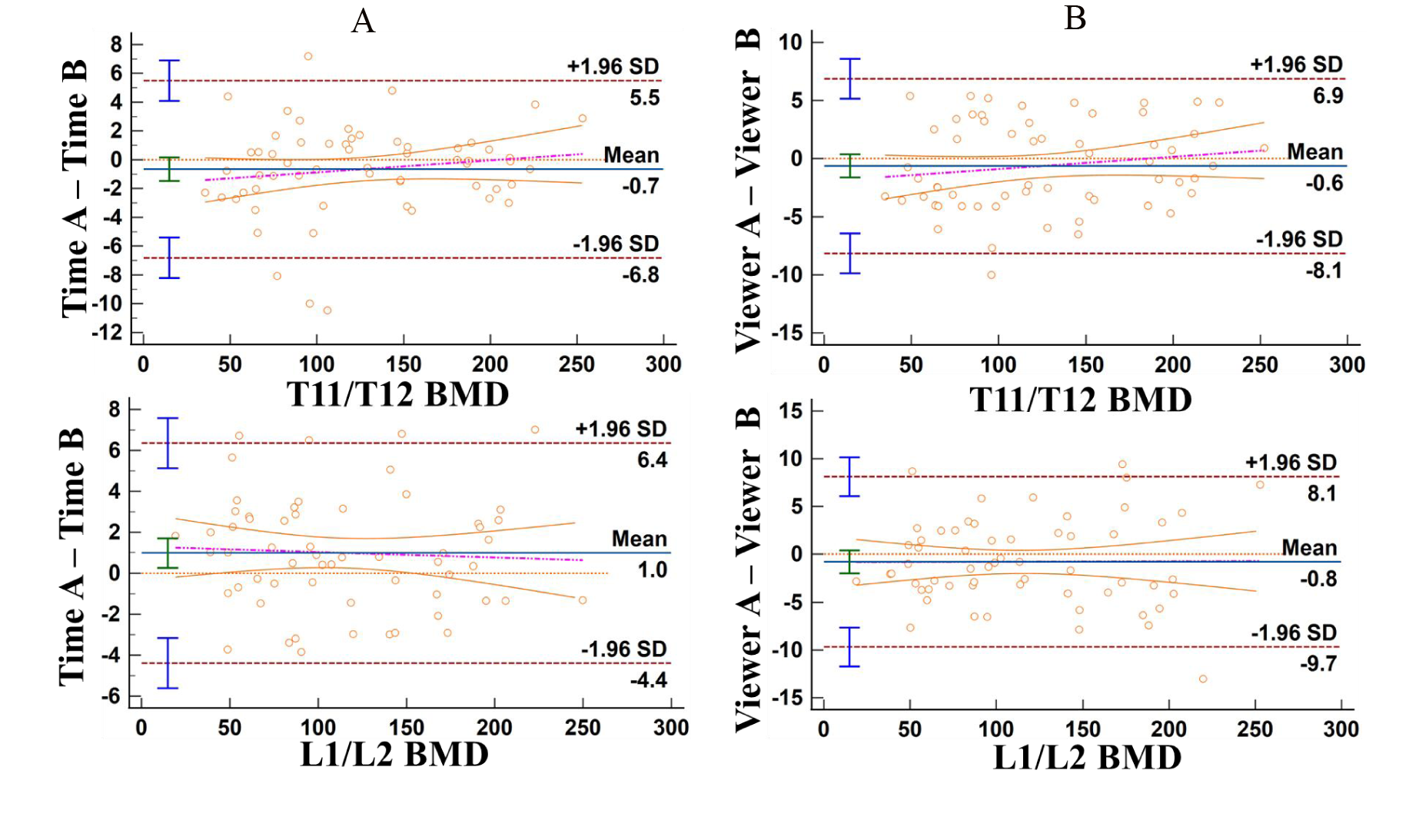


**Supplemental Figure 5**. The performance of thoracic bone mineral density (TBMD) in defining osteopenia and osteoporosis based on 128 and 91 mg/cm^3^ in population two.


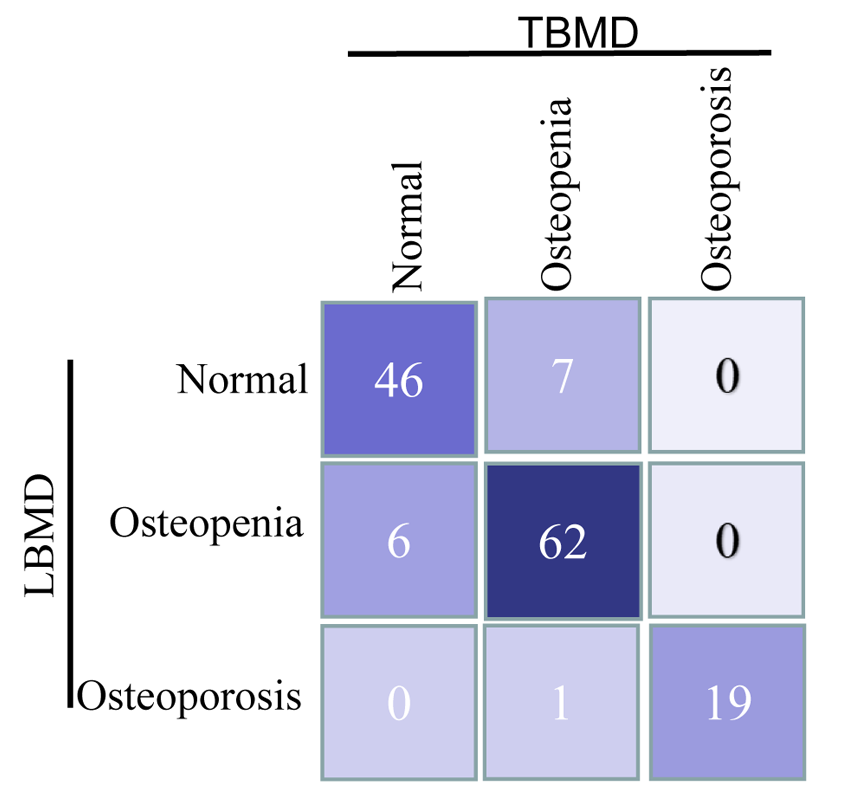


**Supplemental Figure 6**. The performance of translated thoracic bone mineral density (TTBMD) in defining osteopenia and osteoporosis based on 120 and 80 mg/cm^3^ in population two.


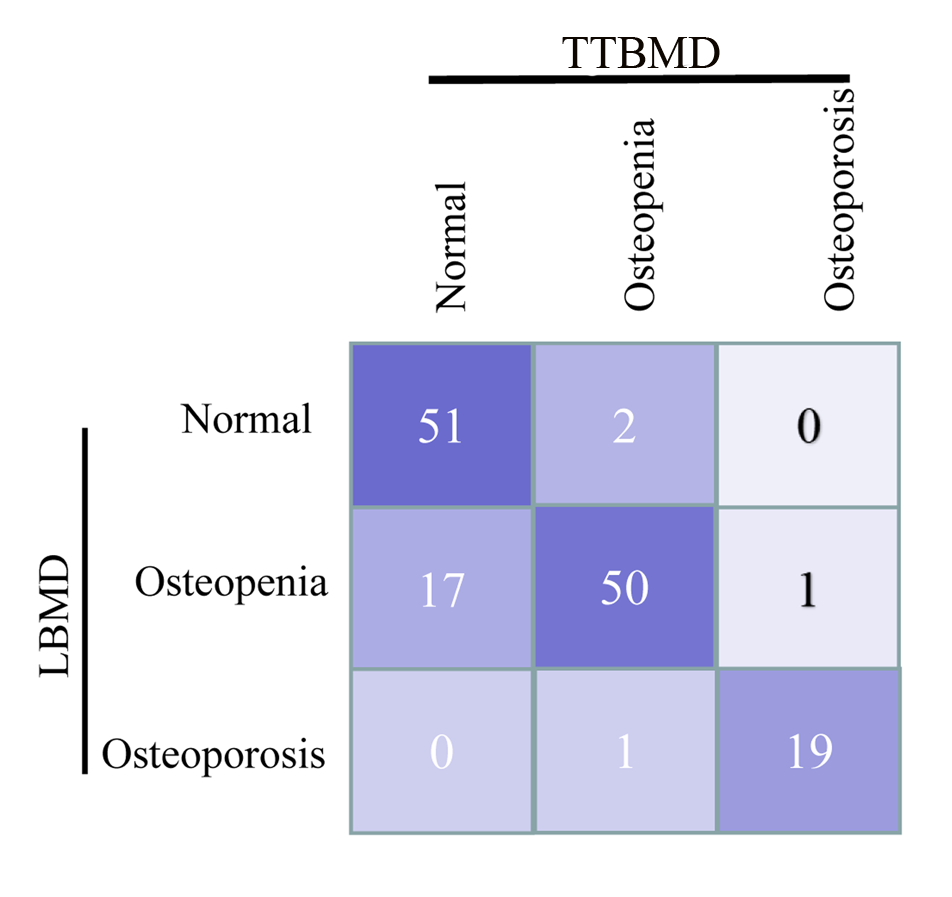


**Supplemental Figure 7**. The association between low bone mass and severe vertebral compression fracture. Cumulative hazards of vertebral compression fracture stratified by osteopenia and osteoporosis based on threshold method (A) and translated thoracic vertebral bone mineral density (B). A: normal: TBMD> 128 mg/cm^3^; osteopenia: TBMD 91-121 mg/cm^3^; osteoporosis: TBMD < 91 mg/cm^3^. B: normal: TTBMD > 120 mg/cm^3^; osteopenia: TBMD 80-120 mg/cm^3^; osteoporosis: TBMD < 80 mg/cm^3^.


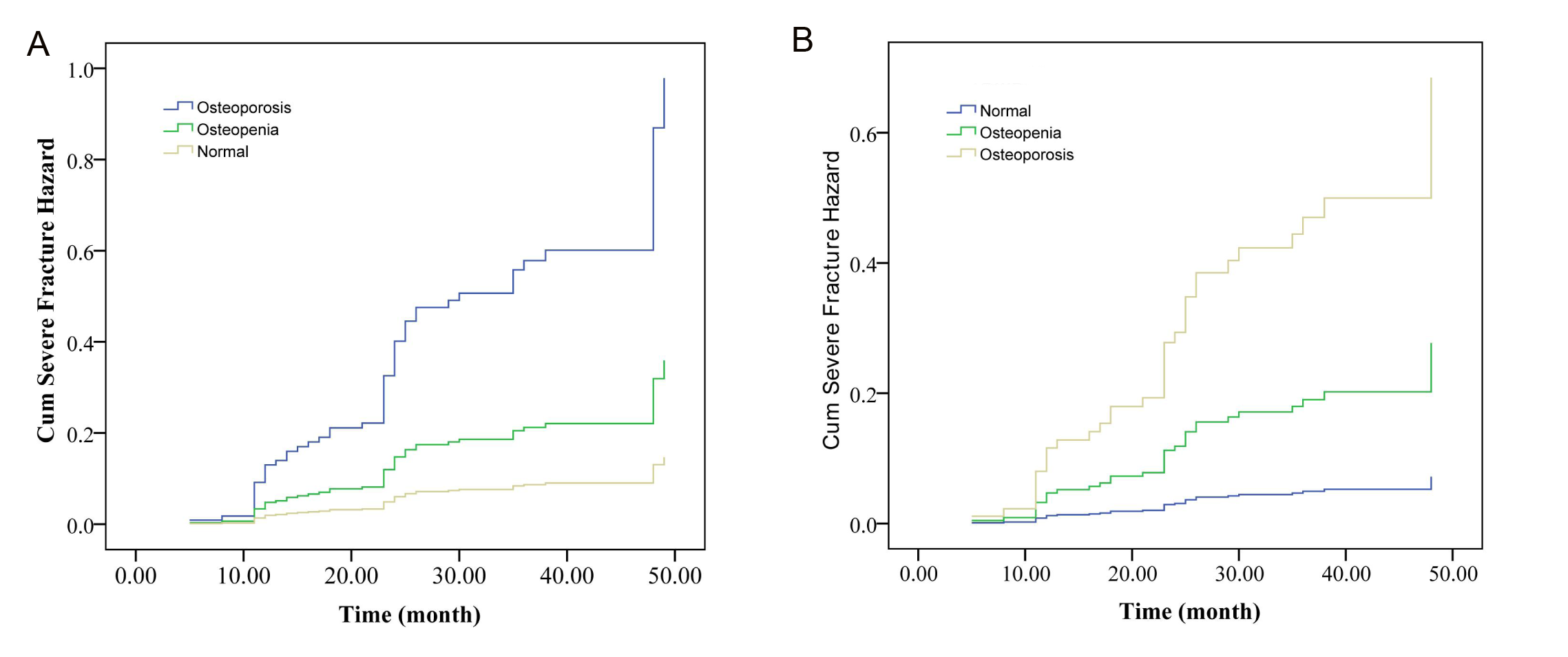

Supplement: Supplementary file 1 — Supplementary Material 1 [file 12877_2024_4737_MOESM1_ESM.docx]
